# Seed priming approaches for climate-resilient agriculture

**DOI:** 10.1093/jxb/eraf440

**Published:** 2025-10-13

**Authors:** Gholamreza Gohari, Alexandros Spanos, Andreas Ioannou, Ioanna Efstathiou, Sima Panahirad, Zsuzsanna Kolbert, Vasileios Fotopoulos

**Affiliations:** Department of Agricultural Sciences, Biotechnology and Food Science, Cyprus University of Technology, Limassol 3603, Cyprus; Department of Horticultural Sciences, Faculty of Agriculture, University of Maragheh, Maragheh 551877684, Iran; Department of Agricultural Sciences, Biotechnology and Food Science, Cyprus University of Technology, Limassol 3603, Cyprus; Department of Agricultural Sciences, Biotechnology and Food Science, Cyprus University of Technology, Limassol 3603, Cyprus; Department of Agricultural Sciences, Biotechnology and Food Science, Cyprus University of Technology, Limassol 3603, Cyprus; Department of Landscape Engineering, Faculty of Agriculture, University of Tabriz, Tabriz 5166616471, Iran; Department of Plant Biology, University of Szeged, Közép fasor 52., Szeged H6726, Hungary; Department of Agricultural Sciences, Biotechnology and Food Science, Cyprus University of Technology, Limassol 3603, Cyprus; Sorbonne University, France

**Keywords:** Climate change, nanocarrier, nanodelivery, nanopriming, sustainable agriculture

## Abstract

Extreme weather events linked with climate change are increasingly affecting global crop production, emphasizing the need to develop and optimize efficient and biosafe technologies with stress-alleviating effects. Seed priming, a pre-sowing treatment that improves seed performance under stress conditions, has emerged as a promising approach for sustainable agriculture. The current review explores latest findings in seed priming techniques, including hydropriming, osmopriming, biopriming, and nanopriming, highlighting their role in enhancing plant resilience against abiotic stress due to climate change. We discuss the physiological, biochemical, and molecular mechanisms underlying priming-induced resilience against abiotic stress. In this concept, priming techniques, with a particular focus on nanopriming, could be exploited as unique stress mitigating practices, with potential for incorporation in sustainable crop management approaches. Nanopriming utilizes nanoparticles to enhance plant resilience to subsequent stress conditions. This strategy can be further improved by utilizing smart nanocarrier systems with distinctive properties, such as being bio-based, biodegradable, biocompatible, non-toxic, with capability to carry a vast array of compounds (e.g. hormones, amino acids, nutrients, essential oils), leading to their sustained and slow release. This innovative approach involves pre-treating seeds to enhance their germination and growth, making them more adaptable to adverse weather conditions.

## Teaser

Seed priming, which represents a pre-sowing treatment that improves seed performance, can be further optimized with the use of advanced nanomaterials and is emerging as a promising approach for sustainable agriculture.

## Introduction

Climate change refers to a prolonged and substantial alteration in climate indicators, such as rainfall, temperature, wind, and snowfall patterns (Pope *et al*., 2022), influenced by both anthropogenic activities and natural factors (Harvey *et al*., 2023). These driving forces have rendered climate change as one of the most urgent challenges of the 21st century. Efforts have been focused on increasing and sustaining crop production in alignment with Goal 2: Zero Hunger of the United Nations’ Sustainable Development Goals (FAO, 2024). This global initiative seeks to eradicate hunger, achieve food security, improve nutrition, and promote sustainable agriculture by 2030. To enhance cereal yield stability, the entire growth cycle from seed to harvest must be considered, with strategies addressing multiple developmental stages (Triboi and Triboi-Blondel, 2002).

In this context, approximately half a million documents have thus far addressed climate change. Considering the key themes in plant stress research, drought, heat, and salinity stress are the central topics considered (Giordano *et al*., 2021; Dos Santos *et al*., 2022; Yadav *et al*., 2022). Such topics are expected, due to the fact that the major impacts of climate change are manifested through an elevation in CO_2_ and increases in temperature and salinity (Yeo, 1998; Chaudhry and Sidhu, 2022). In plant cells, specifically, the levels of reactive oxygen species (ROS), antioxidants, phytohormones, and osmoprotective molecules are critical predictors with respect to stress resilience under changing climate conditions (Hasanuzzaman *et al*., 2020; Hendrix *et al*., 2025). Furthermore, the role of molecular breeding in stress tolerance is highlighted by the importance of genomics, transcriptomics, and CRISPR-Cas9 editing (Razzaq *et al*., 2021; Ali *et al*., 2022). Regarding iconic crop species known for their agricultural, cultural, and economic importance, crop-specific studies on rice, tomato, wheat, maize, and grapevine have been a major focus of research (Ghosh and Padaria, 2025). The primary goal of these studies is to enhance the adaptability of these crops to environmental changes. Among the strategies to enhance resilience, the integration of biostimulants and nanotechnology has emerged as a promising approach for sustainable solutions and climate adaptation. Ensuring climate resilience in crop production is crucial for safeguarding food systems, protecting livelihoods, and promoting sustainable development in the face of climate change (Srivastav *et al*., 2021; Wijerathna-Yapa and Pathirana, 2022). Among the various approaches, seed priming has garnered significant attention for its ability to prepare plants to withstand subsequent stress conditions (Ben Youssef *et al*., 2025; Dolu *et al*., 2025).

Seed priming is a pre-sowing technique that enhances germination and early seed growth by hydrating seeds to a level that initiates metabolic processes, without full radicle emergence. This controlled rehydration activates pre-germinative metabolism while preventing complete germination (Paparella *et al*., 2015; Gohari *et al*., 2024a, b). Seed priming provides numerous benefits, including improved germination rate and uniformity, enhanced stress tolerance, increased vigor and yield, faster maturity, and cost-effectiveness (Chandel *et al*., 2024; Choudhary *et al*., 2024).

**Fig. 1.**
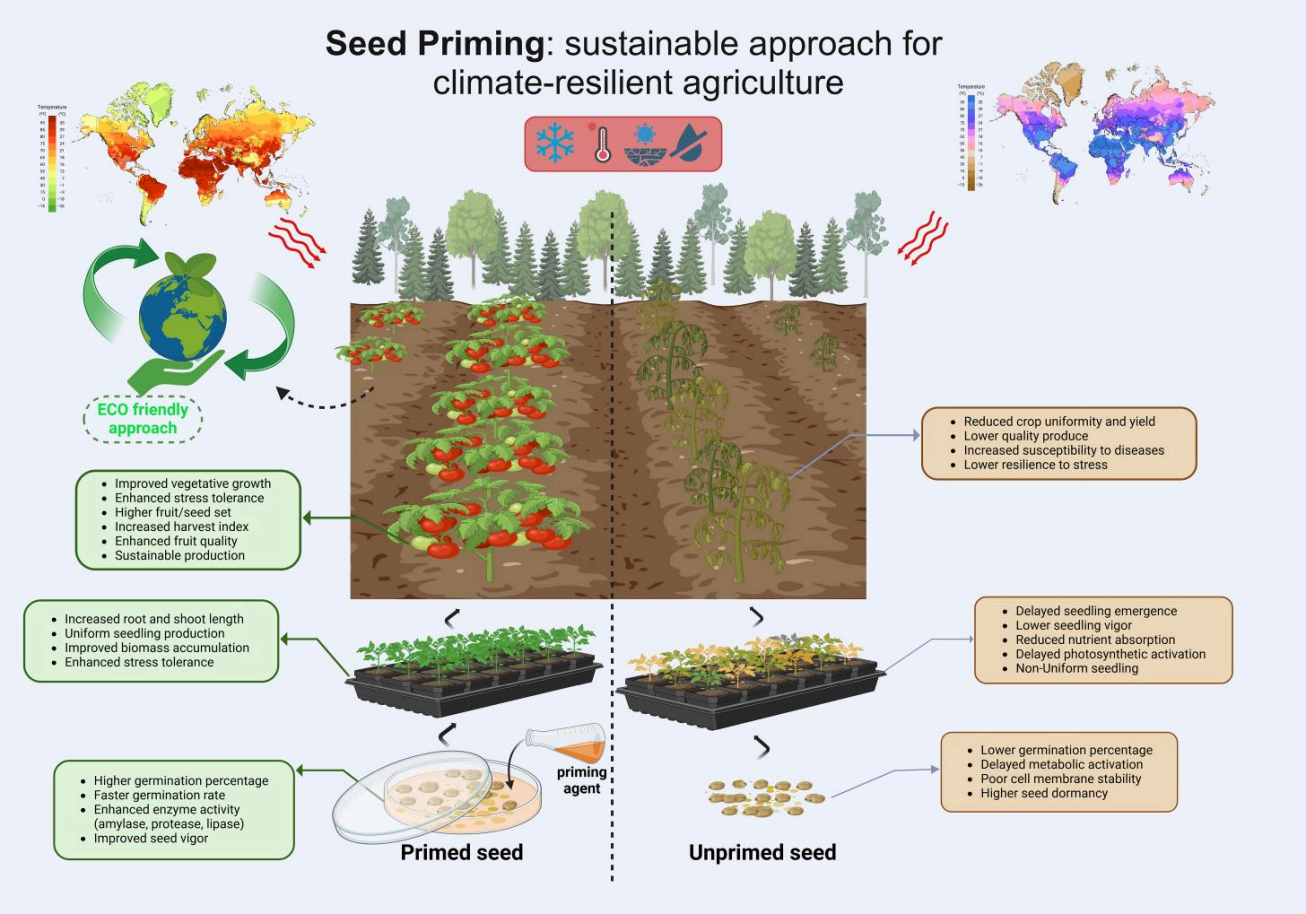
Seed priming as an effective strategy for sustainable and climate-resilient agriculture. The model illustrates the impact of seed priming on plant growth, stress tolerance, and agricultural productivity. Seed priming enhances germination percentage, enzyme activity, and seed vigor, leading to improved seedling establishment, uniform growth, and higher biomass accumulation. This results in increased vegetative growth, better stress resilience, and higher fruit yield with improved quality. In contrast, unprimed seeds exhibit delayed germination, poor seedling vigor, and lower tolerance to environmental stress, ultimately reducing crop uniformity and productivity. Created in BioRender. Gohari, G. (2025) https://BioRender.com/j85k199.

Given the critical impact of climate change on food security, the present review explores seed priming as a strategy for climate-resilient agriculture, focusing on its physiological, biochemical, and molecular mechanisms. It examines various priming techniques (e.g. hydropriming, osmopriming, chemical priming, biopriming, nutrient priming, and nanopriming) and their role in enhancing stress tolerance against drought, salinity, extreme temperatures, and pollutants. The review also highlights recent advancements in nanotechnology for smart delivery systems, evaluates challenges related to efficiency, scalability, and storage, and discusses future directions, including genetic interventions and multi-stress priming. By synthesizing recent findings, this article aims to provide insights for optimizing seed priming applications in agriculture (Fig. 1).

## Understanding seed priming: mechanisms and benefits

The benefits of seed priming have been demonstrated across a wide range of plant species (Ben Youssef *et al*., 2025; El-Shazoly *et al*., 2025). Controlled hydration of seeds is the key concept of priming, but the relevant germination processes are halted before radicle emergence. As in the case of all metabolic processes, the efficiency of seed priming is linked to the type of priming (Tan *et al*., 2025). Regarding the physiological basis of seed priming, metabolic activationsuch as the increased activity of enzymes involved in germination (e.g. amylase, protease, and lipase) and enhanced respiratory activityserves as a key indicator of seed germination following priming treatments (Cao *et al*., 2019; Johnson and Puthur, 2021). The metabolic activation in seeds begins with imbibition, which triggers a series of metabolic processes essential for germination. This activation involves the restoration of cellular metabolism, the balancing of redox states, and the induction of enzymatic activities, including those associated with the TCA cycle and the electron transport chain (Rosental *et al*., 2014).

Furthermore, priming is also associated with repair mechanisms, allowing for the repair of DNA damage that may have accumulated during seed storage (Forti *et al*., 2020; Kiran *et al*., 2020). DNA repair is one of the key mechanisms in the activation of pre-germination metabolism after priming. Following imbibition, oxidative stress can cause extensive DNA damage due to the uncontrolled accumulation of ROS. As discussed by Paparella *et al*. (2015), base-excision repair (BER) and nucleotide-excision repair (NER) pathways are induced in order to maintain the integrity of the genome, and subsequently to ensure successful germination. Studies have demonstrated that transcription-coupled nucleotide-excision repair facilitates the selective removal of DNA lesions from actively transcribed genes, thereby enhancing seedling viability (Kimura and Sakaguchi, 2006; Waterworth *et al*., 2010). The precise balance between DNA repair and oxidative stress responses is critical for the effects of priming in maintaining seed longevity (Paparella *et al*., 2015). In addition, germination and seedling establishment in *Medicago truncatula* have been enhanced through hydropriming and biopriming, which activate DNA repair pathways and antioxidant defenses. Key genes involved in the BER pathway, such as *OGG1* (*8-OXOGUANINE GLYCOSYLASE*) and *FPG* (*FORMAMIDOPYRIMIDINE-DNA GLYCOSYLASE*), were up-regulated (Forti *et al*., 2020).. These genes play a critical role in repairing oxidative DNA damage that accumulates during seed storage and imbibition. Simultaneously, the expression of antioxidant genes, including *SUPEROXIDE DISMUTASE* (*SOD)* and *ASCORBATE PEROXIDASE* (*APX)*, was also up-regulated following hydropriming. As a result, oxidative stress is mitigated, and genomic integrity is preserved in *Medicago truncatula* primed seeds (Forti *et al*., 2020). Interestingly, Kiran *et al*. (2020) reported how NaCl priming (using controlled rehydration with 320 mM NaCl) modulated oxidative stress and activated the key repair pathways during seed germination of *Solanum melongena*. Critically, it was shown that the expression of *KU70*, a pivotal component of the non-homologous end joining repair pathway, was found to be considerably up-regulated in seeds that had been primed with 320 mM NaCl. This priming facilitated the repair of double-stranded DNA breaks during the early stages of germination.

Priming helps repair and maintain membrane integrity and functionality, which are essential for processes like cell expansion and division (Paparella *et al*., 2015; Biswas *et al*., 2023). As previously reported, the oxidative stress and lipid peroxidation levels are reduced following the priming event (Savvides *et al.,* 2016). Such changes have been positively correlated with membrane integrity. Furthermore, the process of priming has been demonstrated to stabilize mitochondrial membranes and promote the synthesis of protective proteins, such as heat shock proteins (HSPs) and late embryogenesis abundant (LEA) proteins. This process (stabilization of mitochondrial membrane) serves to minimize leakage and ensure efficient metabolism within the cell (Varier *et al*., 2010). Stress-related mechanisms, including osmoprotectant accumulation and antioxidant defense, are also modulated and regulated through priming. The accumulation of compatible solutes, such as proline and glycine betaine, helps protect cellular structures in plants under stress conditions. Additionally, key components of the antioxidant systems, such as the activities of antioxidant enzymes, are up-regulated following priming (Savvides *et al.,* 2016;). Regarding cell cycle activation, priming triggers the cell cycle, preparing cells for division and elongation once germination continues (Varier *et al*., 2010; Rosental *et al*., 2014). Interestingly, pre-treatment of Arabidopsis seeds with mimosine, a known cell cycle inhibitor, successfully enhanced seed viability, suggesting that the use of cell cycle inhibitors may offer an improved method of seed priming (Sano and Seo, 2019).

## Update on seed priming techniques towards induced stress tolerance

Several seed priming techniques have been developed and optimized with variable success towards improved growth and development under control conditions, as well as protection against environmental constraints. Interestingly, the various approaches appear to often show functionality against different types of stressors in a ‘horizontal’ manner, revealing cross-induction regulatory elements. This section will summarize main approaches, highlighting latest findings (Table 1).

**Table 1.** Representative studies showcasing the effects of different priming techniques on different plant species

Priming Technique	Crop Species	Stress Condition	Priming Agent	Mode of Action	Reference
Hydropriming	*Vicia faba* L.	C	Water	Increased seedling emergence, fresh weight and seed yield	Damalas *et al*., 2019
Hydropriming	*Sorghum bicolor* L.	D	Water	Improved germination rate index, total seedling length	Dembélé *et al*., 2021
Osmopriming	*Triticum aestivum* L.	D	PEG	Lower ROS formation and lipid peroxidation, higher dry matter and grain yield	Abid *et al*., 2018
Osmopriming	*Amaranthus tricolor* L.	S	PEG	Increased germination rate, improved root anatomical characters	Amalia and Rachmawati, 2023
Osmopriming	*Lactuca sativa* L.	H	K_3_PO4	Better and faster root development, early cell division and elongation	Cantliffe *et al*., 1984
Biopriming	*Trigonella foenum-graecum* L.	C	*Azospirillum*	Increased total biomass, shoot FW and DW	Solouki *et al*., 2023
Biopriming	*Triticum aestivum*	D	*Metarhizium anisopliae*	Improved root growth, morphological traits, yield characteristics	Mim *et al*., 2024
Biopriming	*Oryza sativa* L.	HM	PGRs	Improved final germination and germination rate, enhanced vigour and dry biomass, reduced Cr content in leaves	Singhal *et al*., 2023
Biopriming	*Secale montanum* L.	D	*Bacillus Pseudomonas*	Improved FE, DW, growth indices, and nutrient uptake	Rahnama *et al*., 2023
Chemical Priming	*Triticum aestivum* L.	D	NO and H_2_O_2_	Improved leaf water potential, turgor, relative water content, photosynthetic pigments	Habib *et al*., 2020
Chemical Priming	*Pisum sativum L.*	H	CaCl_2_+SA	Improved germination attributes, seedling growth, FW and DW, chlorophyll and relative water content	Tamindžić *et al*., 2023
Chemical Priming	*Glycine max*	D	GA3+BAP	Up-regulation of drought-related genes (*SOD-CCS, SODB2, BADH2, DREB2A*)	Jaybhaye *et al.*, 2024
Chemical Priming	*Cajanus cajan*	HM	ABA	Improved germination, seedling growth, FW and DW	Rhaman *et al.*, 2020
Chemical Priming	*Triticum aestivum*L.	S	SNP	Improved seed germination, seedling growth, photosynthetic pigments	Ali *et al*., 2017
Chemical Priming	*Arachis hypogaea* L.	D	Melatonin	Early root development, enhanced antioxidant activity and reduced ROS accumulation	de Camargo Santos *et al*., 2024
Chemical Priming (Nutrient Priming)	*Canavalia ensiformis* L.	C	Mo	Induced photosynthetic pigments, improvement in growth and increased biomass	Okla *et al*., 2021
Chemical Priming (Nutrient Priming)	*Oryza sativa* L.	D	B	Improved seedling emergence, leaf appearance and elongation, chlorophyll, water relations and yield	Rehman *et al.*, 2015
Chemical Priming (Nutrient Priming)	*Capsicum annuum* L.	C	Zn-Mo	Improved effectiveness on seed imbibition and germination with better initial absorption	Ochoa-Chaparro *et al*., 2024
Chemical Priming (Nutrient Priming)	*Oryza sativa* L.	D	Se	Enhanced seed germination and seedling growth, increased antioxidants enzymatic activities	Moulick *et al.*, 2017
Nano Priming	*Solanum lycopersicum* L.	C	ZnO+TiO_2_ NPs	Increased germination, seedling vigour and promoted ROS scavengers, such as SOD, POD, and CAT	Sonawane *et al.*, 2021
Nano Priming	*Zea mays* L.	S	Silica NPs	Higher germination rate and seedling vigour index, antioxidant enzymatic activity	Naguib and Abdalla, 2019
Nano Priming	*Oryza sativa* L.	C	ZnO NPs	Increased seed and straw yield, enhanced proline	Waqas Mazhar *et al.*, 2022
Nano Priming	*Arachis hypogaea*	S	GO NPs	Improved seed germination rate and radicle emergence	Yan *et al*., 2024
Nano Priming	*Valerianella locusta* L.	S	Chitosan-melatonin NPs	Increased biomass, photosynthetic pigments and antioxidant enzymatic activities	Gohari *et al.*, 2024a, b
Nano Priming	*Oryza sativa*	D	SiO_2_ NPs	Triggered ROS production and activated drought responsive genes	Kang *et al*., 2025

(C: Control; D: Drought; S: Salinity; H: Heat; HM: Heavy Metal)

### Hydropriming

Hydropriming is the most commonly used seed priming technique for improving germination, where the seeds are soaked in water for a certain duration, depending on the cultivar, without allowing the radicle to appear. Next the seeds are dried to their initial weight and can either be used immediately or stored for future use (Paparella *et al*., 2015). The pattern of water absorption by the seeds consists of three main phases. The first phase is characterized by quick imbibition of water due to the low water potential within the seeds. During this first event, the hydration of different cellular components can restore membrane integrity and activate enzymes that participate in seed germination. During the middle lag phase, while water uptake reaches a plateau, metabolic activity is increased, with mitochondrial biosynthesis. In the last phase, water uptake is increased, with cell division and expansion leading to germination of the radicle. However, priming should stop before the completion of the second lag phase to keep the process reversible (Adhikari *et al*., 2021). The main disadvantage of hydropriming is that the water absorption by seeds could be uncontrolled, and this may lead to unequal metabolic activation within seeds, followed by unsynchronized radicle emergence. For that reason, it is very important to determine the right volume of water required to hydrate seeds, but also to assign the accurate treatment duration and temperature (Dawadi and Karki, 2023). Hydropriming has been reported to improve germination percentage, germination rate index and total seedling length, contributing to increased seedling emergence and yield under both optimal and stress conditions, allowing plants to better establish themselves and withstand abiotic stresses (Dembélé *et al*., 2021). Furthermore, Tamindžić *et al*. (2023) reported that hydroprimed pea seeds (*Pisum sativum* L.) resulted in increased tolerance to heat stress, including improvements in mean germination rate, seedling vigor index, shoot and root elongation rate, as well as membrane stability index. Overall, this method is considerably low-cost, efficient, easy to implement, and particularly beneficial for improving crop productivity without any additional chemical inputs.

### Osmopriming

In the osmopriming method, seeds are immersed in an osmotic solution of lower water potential. Various chemicals, such as polyethylene glycol (PEG), mannitol, sorbitol, and inorganic salts like CaCl_2_, KNO_3_, KCl_3_, K_3_PO_4_ and NaCl, have been investigated as potentially effective osmopriming agents (Koushal *et al*., 2024). This kind of immersion allows the seeds to absorb water slowly, and prolong their imbibition stage, leading to a gradual advancement of pre-germination procedures and metabolic activities. This parameter is vital, as it helps regulate water uptake, limiting ROS accumulation, and reducing oxidative damage to several cellular components (Paparella *et al*., 2015; Saha *et al*., 2022). Among the chemicals used, PEG is the most commonly used priming agent. PEG is a non-ionic polymer that does not chemically interact with the seeds. Furthermore, its large molecular size prevents it from penetrating the seed, therefore avoiding the risk of penetrating seed tissues and causing potential cytotoxic effects (Amalia and Rachmawati, 2023). Osmopriming of crop seeds with PEG prior to exposure to abiotic stress has improved germination and seedling emergence (e.g. Zhang *et al*., 2015; Lei *et al*., 2021; Ma *et al*., 2024; Wang *et al*., 2024). Nevertheless, the high viscosity of PEG can limit oxygen transfer within the priming solution, and for that reason, it is highly recommended to use an open aeration system to minimize this kind of event. However, these systems are costly, especially for large scale applications (Paparella *et al*., 2015; Ma *et al*., 2024; Wang *et al*., 2024).

### Halopriming

Priming with inorganic salts is also referred to as halopriming; however, this method works as a preventive strategy to improve the resilience of plants to several abiotic stresses by activating stress-related genes, enzymes and metabolic pathways that help the plants to protect cellular tissues and improve homeostasis (Biswas *et al*., 2023; Pagano *et al*., 2023a). A recent study by Tamindžić *et al*. (2023) reported that pea seed priming with CaCl_2_ application increased tolerance to heat stress, linked with better and faster root development in soil during heat stress conditions. Similarly, Paul *et al*. (2025) used low concentrations of NaCl (50 mM) as a seed priming agent on moderately salt-tolerant chickpea (*Cicer arietinum*), subsequently exposed to salinity stress (NaCl 50 mM). The primed seeds displayed enhanced stress tolerance, with an increase in reduced ascorbate and glutathione contents, along with increased activity of glutathione reductase and dehydroascorbate reductase. However, it should be noted that priming with salts like NaCl can lead to endogenous toxicity problems, due to the accumulation of sodium and chloride ions in seeds, which may negatively affect germination and health of seedlings (Cano *et al.,* 1991). It is therefore very important to determine the optimal concentration of the treatment and priming duration, based on the specific type of salt used and plant species.

### Biopriming

Biopriming is an established seed inoculation method that involves soaking seeds in water containing beneficial microorganisms, such as fungi and plant growth-promoting bacteria (Cardarelli *et al*., 2022). This kind of treatment enhances germination rate and uniformity at similar levels compared with other seed priming methods (Waqas *et al*., 2019). Moreover, various strains of bacteria and fungi, like *Bacillus* spp., *Enterobacter* spp., *Pseudomonas* spp., and *Trichoderma* spp. can colonize the rhizosphere of plants, support plant growth and tolerance by enhancing metabolic activities, and make vital nutrients more soluble and bioavailable, particularly at the crucial early stages of germination (Fiodor *et al*., 2023). However, its main advantage is protecting seeds and young seedlings against soil and seed-borne pathogens, as well as various abiotic stresses. More specifically, such microorganisms help plants to improve their tolerance by triggering defense mechanisms like induced systemic resistance, while also regulating plant hormonal profile and related gene expression (Hadj Brahim *et al*., 2022; Solouki *et al*., 2023). Biopriming has been particularly effective towards the amelioration of water deficit effects in plants. For example, *Metarhizium anisopliae* (*MA*), a fungal endophyte known for its resistance to unfavorable environmental conditions, was used by Mim *et al*. (2024) as a seed priming agent to mitigate the adverse effects of drought stress on wheat (*Triticum aestivum*). *MA*–primed seeds were tested under 20% (moderate drought) and 10% (severe drought) soil moisture content. Interestingly, under both stress levels, *MA* enhanced root growth, morphological traits, yield characteristics, and photosynthetic activity, while boosting enzymatic antioxidant activity and reducing stress markers such as malondialdehyde (MDA) and hydrogen peroxide (H_2_O_2_) content. A similar study by Naz *et al*. (2024) showed that *MA*-primed barley (*Hordeum vulgare*) seeds under drought stress enhanced biomass, leaf traits, photosynthetic pigments and antioxidant enzymatic activities, as well as non-enzymatic antioxidants, such as ascorbate and glutathione content. *MA* significantly reduced stress markers, including MDA, H_2_O_2_, methylglyoxal, calcium influx, and lipoxygenase, while increasing potassium and proline content.

Overall, biopriming is a promising, eco-friendly technique for improving crop resilience and productivity, while reducing the need for chemical fertilizers and fungicides, contributing to more sustainable agricultural practices. Nonetheless, it remains unclear how live microorganisms are incorporated into seeds, or how they remain affixed to seed coats as priming agents. A common method in biopriming is hydrating seeds before or after exposing them to a suspension of microorganisms as priming agents. However, the viability, uptake, and mode of action of microbes on primed seeds still need to be determined (Paparella *et al*., 2015; Singh *et al*., 2020). Protective biopolymers and coatings, as well as natural substances like kimchi sauce, could enhance microbial viability through biopriming technology (Chakraborti *et al*., 2022; Tiwari *et al*., 2025). Seed germination, seedling vigor and crop production can be increased by using bio-based carriers like alginate, xanthan gum, gum arabic, and carboxy-methylcellulose, to deliver plant growth-promoting microorganisms through seed biopriming (Pérez-Jaramillo *et al*., 2016; Chin *et al*., 2022). Furthermore, Mitchener *et al*. (2025) studied the effects of biopriming duration, seed input, and storage conditions on *Pseudomonas fluorescens* viability during *Brassica napus* seed germination. According to their results, bacterial viability significantly decreased during long-term dry storage at 21 °C, with counts dropping from 9.8×10⁸ to 5.0×10² CFU per seed over 12 weeks. However, under cooler conditions (4 °C), microbial viability is significantly improved. Furthermore, bacterial survival significantly increased when kimchi substrate was added. Factors affecting biopriming, such as seed drying methods, inoculum preparation, formulation, and storage environment critically influence bacterial survival. In order to optimize biopriming technology, traditional priming methods (e.g. hydropriming and osmopriming) must be integrated with new storage conditions and protective methods like seed coating or using different bacterial nutrient substrate.

### Chemical priming

Chemical seed priming is an established method that utilizes natural or synthetic compounds as priming agents. This technique includes agents like hormones (e.g. melatonin, salicylic acid, gibberellins), polyamines (e.g. spermidine, spermine, putrescine), carbohydrates (e.g. trehalose, sucrose, raffinose) and vitamins (e.g. ascorbate), as well as reactive oxygen–nitrogen–sulfur species (RONSS) donors such as sodium hydrosulfide (NaHS), sodium nitroprusside (SNP), and the novel chimeras NOSH and NOSH-aspirin (Savvides *et al*., 2016; Antoniou *et al*., 2020; Gohari *et al*., 2024a, b; Tripathi *et al*., 2024). The mechanisms of action of these chemicals differ based on the specific substance utilized, but they frequently include the regulation of ion balance, activation of antioxidant mechanisms, expression of genes responsive to stress, and the accumulation of osmoprotectants (Antoniou *et al*., 2020; Habib *et al*., 2020; Jatana *et al*., 2024). For example, the application of H_2_O_2_ or nitric oxide (NO) donors can trigger the production of antioxidant enzymes, thereby enhancing the capacity of the plant to neutralize ROS (Habib *et al*., 2020). In contrast, melatonin serves as a direct scavenger, and can initiate antioxidant responses (Khan *et al*., 2019; de Camargo Santos *et al*., 2024). Similarly, Doddagoudar *et al*. (2023) reported that using salicylic acid as a chemical priming agent on rice seeds resulted in increased germination rate and seedling vigor, while enhancing antioxidant enzymatic activities (i.e. CAT, SOD, and POD) under cold stress conditions. Furthermore, pigeon pea (*Cajanus cajan*) seeds primed with 100 µM abscisic acid (ABA) showed improved germination when exposed to cadmium stress. This improvement was attributed to ABA-dependent inhibition of the biosynthesis of enzymes responsible for endosperm wall degradation, thereby influencing seed germination (Sneideris *et al*., 2015). Although chemical priming can lead to substantial improvements in crop performance, it is essential to optimize the process carefully to prevent phytotoxicity, and to determine the suitable concentration and duration of treatment for the specific crop and stress conditions (Savvides *et al*., 2016).

### Nutrient priming

Nutrient priming, or nutripriming—a specific type of chemical priming—involves soaking seeds in solutions containing necessary macro- or micronutrients to increase germination, seedling resilience, and stress tolerance (Houmani *et al*., 2024). This technique combines the biochemical benefits of seed priming with the nutritional effect of the applied nutrient, contributing a low-cost, effective, and sustainable process (Johnson and Puthur, 2021). The key nutrients that are usually used are zinc, phosphorus, boron, molybdenum, and manganese, intending to increase the nutrient content of the seeds, promoting germination, seedling growth, and root system development (Ochoa-Chaparro *et al*., 2024). Furthermore, an adequate supply of these nutrients assists protein synthesis, cell elongation and expansion, promoting better establishment of the plants, which leads to greater yield and seed quality (Saha *et al*., 2022). A promising approach was followed by Kasana *et al*. (2025), who investigated the potential synergistic effect of combined application of 0.5% Zn and 3 mM glutathione (GSH) on salt stress tolerance in maize seedlings. Interestingly, combined treatments effectively diminished high salinity-induced stress by enhancing germination attributes, seedling emergence and growth, as well as boosting foliar photosynthetic pigments and reducing MDA content. Moreover, this synergistic treatment limited Na^+^ uptake and translocation, while maintaining optimal K^+^/Na^+^ and Ca^2+^/Na^+^ ratios, playing a key role in improving salt tolerance in maize.

### Solid matrix priming

Matrix priming or solid matrix priming (SMP), as a proven priming treatment, includes mixing seeds with solid substrates/materials (e.g. vermiculite, sand, perlite, shale) and water in distinctive proportions to help imbibition of seeds in soil. SMP helps the water balance of seeds and consequently leads to improved germination through enhanced physiological and biochemical activities (Pagano *et al*., 2023b). The solid substrates of SMP lead to a threshold moisture content in seeds due to a high water-holding capacity, but prevent radicle emergence (Wu *et al*., 2019). In fact, SMP materials cause moderate hydration of seeds, acting similar to osmo- or hydropriming if a sufficient amount of oxygen is provided during the hydration process. In addition, SMP is cheaper and easier to execute than chemical priming, and supports the incorporation of any biological agents (e.g. fungicide, insecticide). Likewise, SMP helps protecting seeds against stressors (Manzoor *et al*., 2023). SMP improves seed germination traits in comparison with other priming methods, and increases organic storage substances, several antioxidants and anti-oxidative enzymatic activities, as well as metabolite accumulation, thus representing a useful method to improve plant tolerance to stressors (Wu *et al*., 2019).

### Nanopriming

Nanotechnology, utilizing nanoparticles (NPs) less than 100 nm in size, has been demonstrated to play a promising role in various industries, particularly agriculture (Johnson and Puthur, 2021). Priming with diverse NPs, also known as nanopriming, has been shown to increase germination and seedling development due to the greater penetration properties of NPs, improved stability, and bigger surface area providing closer interactions in biological matrixes, primarily facilitating nutrient and water uptake (Johnson and Puthur, 2021). Under adverse conditions, priming with NPs could enhance plant tolerance to abiotic stress (Nile *et al*., 2022; Pagano *et al*., 2023a; Gohari *et al*., 2024a, b) by stimulating the antioxidant system, increasing the efficiency of photosynthetic electron transport, and sucrose biosynthesis (Johnson and Puthur, 2021). In addition, different genes related to plant stress tolerance are activated, while lowering needs for pesticide and fertilizer application (Ioannou *et al*., 2020; Spanos *et al.,* 2023). Nanopriming efficiently affects the plant lifecycle from germination to yield, as well as food nutritional quality, under normal and stress conditions (Nile *et al*., 2022; Thakur *et al*., 2022). Furthermore, it leads to direct effects in the enhancement of secondary metabolite biosynthesis, with well-recorded plant stress-protecting and health-promoting effects (Nile *et al*., 2022). Nanopriming could entail the direct use of NPs (e.g. metal-based, carbon-based) or engineered NPs based on nanocarriers functionalized with a wide range of compounds (e.g. hormones, amino acids) (Panda and Mondal, 2020; Waqas Mazhar *et al*., 2022). For example, Gohari *et al*. (2024a, b) reported that seed priming with chitosan-melatonin and chitosan-salicylic acid nanoparticles on corn salad (*Valerianella locusta* L.) seeds not only enhanced plant performance, but also increased secondary metabolite content under moderate and severe salt stress conditions, through an increase in naringin, chlorogenic acid, catechin hydrate and *o*-coumaric acid content. Similarly, graphene oxide (a widely used industrial nanomaterial) was applied as a priming agent on peanut (*Arachis hypogaea*) seeds to assess its effects on germination, seedling salinity tolerance, and yield. In this experiment, graphene oxide enhanced seed germination rate and radicle emergence, while boosting carbon and nitrogen metabolism pathways. Transcriptomic and metabolomic analyses linked graphene oxide priming to key pathways involved in photosynthesis, phytohormones, and stress responses (Yan *et al*., 2024).

## Smart technology for delivery of nanopriming agents

Increasing attention is being given to the employment of nanotechnological tools in seed priming approaches. As briefly mentioned above, nanopriming could be applied through two major groups: direct use of active NPs and sustained-release nanocarrier systems. In both groups, NPs are taken up and retained by seed coats. Active NPs have a direct biological effect, and act as stimulant or anti-microbial agent, leading to beneficial effects in plant growth and development (Gohari *et al*., 2024a, b). In sustained-release nanocarrier system (smart nanodelivery system), the system contains active or non-active (passive) NPs with active compound (biological or synthetic) loadings (e.g. nutrients, fungicides, bactericides, essential oils, plant growth regulators) that slowly release the active compounds over time. The latter system results in improved biological impacts and lower toxicity (do Espirito Santo Pereira *et al*., 2021). The controlled release of specific compound concentrations assists in the preservation of soil health (Nile *et al*., 2022). Advanced nanodelivery systems facilitate both passive and active targeted transport and enable controlled release of various active ingredients, thereby enhancing efficiency of seed germination and seedling vigor (Gohari *et al*., 2024a, b). Nanodelivery systems contain NPs made from polysaccharides, lipids, and proteins, with biodegradability, biocompatibility and capacity to react to altered environmental stimuli. The NPs could modify the root volume and beneficial microbial community, particularly in the rhizospheric zone, leading to improved elemental uptake and ultimately increased yield. The key properties of NPs, including chemical nature, size, concentration, as well as soil texture, pH and organic matter content , determine their positive effects in plants (do Espirito Santo Pereira *et al*., 2021). Numerous unique nanocarriers have been engineered to be applied in smart delivery systems as an advanced targeted priming strategy, providing an eco-friendly, next-generation method, leading to higher effectiveness and lower chemical usage, lowering its potential environmental impact for achieving sustainable agriculture (Gohari *et al*., 2021, 2024a, b).

Nanocarriers (depending on their physical and chemical properties) result in promising impacts on crops, such as improved absorption of nutrient elements, photosynthesis, seed germination, and yield and quality of crops and, therefore, plant growth and development. Two major categories relying on the material properties (physical and chemical) qualify for use as either organic or inorganic nanocarriers (Gohari *et al*., 2024a, b). Various nanocarriers with a potential of smart nanodelivery could be exploited in seed priming approaches, such as chitosan, alginate, zein, cellulose, synthetic biopolymers, and lipid NPs. Nowadays, chitosan is widely utilized in nanocarrier systems for agricultural applications with fungicidal properties, antimicrobial activities, and and modulating plant metabolism and defense (do Espirito Santo Pereira *et al*., 2021). Chitosan is broadly adopted as a nanocarrier due to it being bio-based, bio-safe, non-toxic, and biodegradable, holding notable potential for delivering many compounds (Gohari *et al*., 2024a, b). This biocompatibility was recently highlighted in a report by Giovannini *et al*. (2025), where tomato seeds were coated with salicylic acid encapsulated with chitosan, and in combination with arbuscular mycorrhizal fungi (AMF) inoculation, led to improved tolerance to water deficit and salinity, that was largely attributed to major transcriptional and metabolic rewiring. However, the concentration of chitosan is crucial, as lower concentrations may overstimulate seeds by enhancing water absorption, whereas higher concentrations can inhibit seed germination by eliciting physiological responses leading to cell apoptosis (Sen and Das, 2024).. The preparation protocols of these NPs do not normally contain organic solvents, while their physico-chemical proprieties (i.e. higher solubility, protection against degradation, and bioavailability) result in lower needs for active compounds and reduced toxicity effects (do Espirito Santo Pereira *et al*., 2021).

Graphene oxide (a carbon-based nanocarrier) and carbon quantum dots have been additionally identified as favorable candidates for smart delivery of several compounds (e.g. Proline, Putrescine) to crops. By using this method, stress tolerance of crops can be enhanced by improving enzymatic and non-enzymatic antioxidants, due to small size of carbon quantum dots and capability of transporting biomolecules. Such beneficial effects were shown in a recent report by Zhang *et al*. (2025), where graphene oxide seed coatings resulted in improved maize seedling growth, linked with an increase in carbon and nitrogen metabolism. Clay-based carriers could also act as promising vehicles for delivery of several compounds to plants (Gohari *et al*., 2024a, b). In recent research by Abrahimi *et al*. (2023) and Kumari *et al*. (2024), coating rapeseed and rice seeds with bentonite and montmorillonite-based materials, respectively, resulted in improved germination percentage, vegetative growth, and yield plants.

## Molecular and physiological basis of priming-induced stress tolerance

Priming, as a sustainable crop management practice, enhances plant tolerance to stress conditions by inducing short- and long-term stress memory. This process involves physiological, cellular, and molecular changes that prepare plants for future stress exposure, including the production and activation of signaling molecules, enhancement of metabolic processes, and promotion of protein synthesis, enzymatic activity, and antioxidant responses. Furthermore, it supports DNA repair, cell division, and ATP and osmolyte synthesis, depending on stress type and intensity. Additionally, priming increases mitochondrial respiration and facilitates mitochondrial repair, reactivation, and biogenesis (Thakur *et al*., 2022; Biswas *et al*., 2023; Jatana *et al*., 2024). By using high-throughput omics technologies, researchers have been able to decipher key molecular mechanisms related to seed priming, such as DNA methylation and histone acetylation (Corbineau *et al*., 2023; Hernandez *et al*., 2024). Previous studies on different plant species, such as alfalfa, rice and wheat, have shown the up-regulation of genes linked to translation and epigenetic regulation during priming (Yacoubi *et al*., 2011; Fercha *et al*., 2013; Cheng *et al*., 2017). This molecular modification may contribute to enhanced plant resilience by creating a form of stress memory through epigenetic modifications.

The phenomenon of priming directly affects plant physiological performance, manifested by influencing cell division, plasma membrane fluidity, ion transport by enhancing Na^+^/H^+^ antiporter activity [mediated by *Nax1* (*TmHKT1;4-A2*) and *Nax2* (*TmHKT1;5-A*)], resulting in Na^+^ exclusion, and H^+^-ATPase activity [mediated by high-affinity K+ transporter 5 (*HAK5*) and K^+^ uptake permease 7 (*KUP7*)], which help stabilize membranes. It also enhances transporter activity (HKT1;4) that reduces Na^+^ translocation to leaves, thereby improving osmotic regulation (Ibrahim, 2016; Biswas *et al*., 2023). Additionally, priming promotes the uptake of essential minerals like Zn, K, Ca, and P, optimizing nutrient use efficiency (Thakur *et al*., 2022). The positive changes in the elemental uptake and export decrease the plant osmotic potential and increase the water uptake (Ibrahim, 2016; Jatana *et al*., 2024), further facilitated by regulating plasma membrane (PIP) and tonoplast (TIP) aquaporins (Paul *et al*., 2022: Thakur *et al*., 2022). Furthermore, priming leads to increase in enzymatic activities, causing mobilization of stored nutrients (Marthandan *et al*., 2020; Biswas *et al*., 2023). In addition, priming has been shown to induce compatible solute accumulation, osmoprotection, and macromolecule preservation (Marthandan *et al*., 2020), linked with the induction of stress-responsive genes related to osmolyte production [delta-1-pyrroline-5-carboxylate synthetase (*P5CS*), pyruvate dehydrogenase (*PDH*) and 3-hydroxybutyrate dehydrogenase 1 (*BADH1*)] (Biswas *et al*., 2023; Jatana *et al*., 2024). In terms of carbohydrate metabolism, priming increases chlorophyll content and stomatal conductance, and decreases transpiration rate under subsequent stressful conditions (Zulfiqar, 2021; Biswas *et al*., 2023), leading to better photosynthetic efficiency and chlorophyll fluorescence parameters, and PSII efficiency (Zulfiqar, 2021).

Stress conditions lead to generation and accumulation of ROS, with destructive effects via lipid peroxidation and protein degradation, as well as nucleic acid alternations; however, this damage could be neutralized through priming-induced effects (e.g. antioxidant system activation) (Savvides *et al*., 2016; Paul *et al*., 2022). ROS homeostasis, a fundamental event after priming, controls H_2_O_2_ metabolism that controls oxidative signaling in association with other ROS and signaling molecules (e.g. NO and hydrogen sulfide, H_2_S), (Thakur *et al*., 2022; Gohari *et al*., 2024a, b). In fact, priming-induced osmotic regulation leads to ROS neutralization by enzymatic (e.g. CAT, SOD, APX, POD, GP) and non-enzymatic (e.g. phenols, anthocyanin, flavonoids) antioxidants (Jatana *et al*., 2024), linked with molecular cross-talk between ROS and phytohormones (Savvides *et al*., 2016; Thakur *et al*., 2022).

Major hormonal and metabolic reprogramming events are regulated via priming processes. Priming alters plant hormonal balance and stress adaptation by triggering the biosynthesis of hormones, including gibberellins, auxins, and polyamines, which supports root growth and development at first stages (Marthandan *et al*., 2020; Zulfiqar, 2021; Thakur *et al*., 2022; Biswas *et al*., 2023). Genes encoding ABA biosynthetic enzymes and transcription factors such as Transcriptional Activator of ABA-Responsive 1 (*TRAB1*), WRKY DNA-binding protein 71 (*WRKY71*), polyamine-metabolic enzymes such as S-Adenosylmethionine decarboxylase (*SAMDC*), Spermidine synthase (*SPDS*), Spermine synthase (*SPMS*), Diamine oxidase (*DAO*) and polyamine oxidase (*PAO*) are additionally up-regulated by priming, leading to osmoprotection (Biswas *et al*., 2023; Jatana *et al*., 2024). Furthermore, priming enhances calcium levels in the cytosol, important for cell functions, signal amplification, and regulation of transcription factors, leading to faster and resilient immune responses of stressed plants (Biswas *et al*., 2023; Jatana *et al*., 2024).

**Fig. 2.**
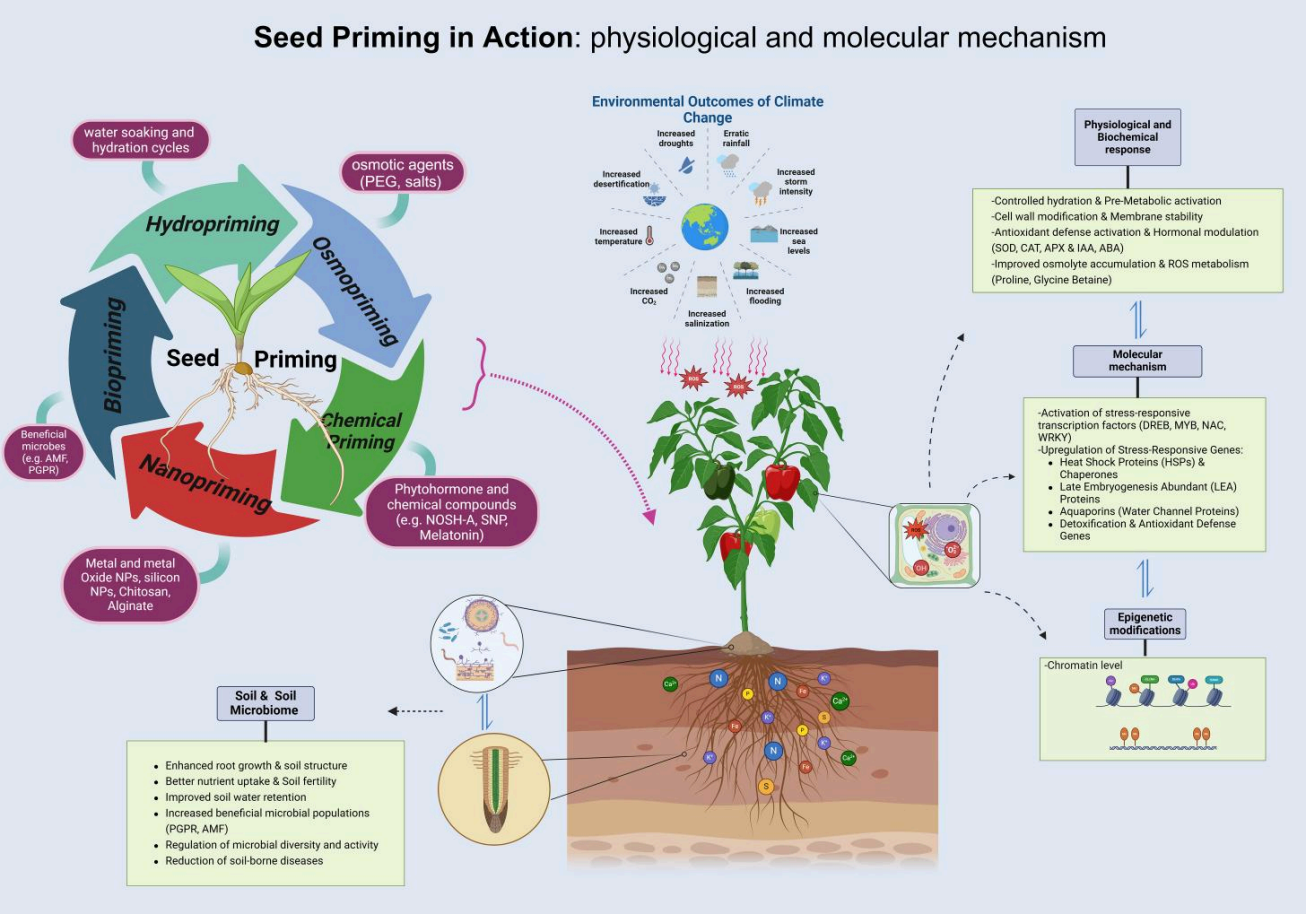
Seed priming mechanisms. Several seed priming techniques like hydropriming, osmopriming, biopriming, chemical priming and nanopriming have been developed and optimized with variable success towards improved growth and development under control conditions, as well as protection against environmental constraints. Seed priming influences multiple physiological and biochemical processes, such as pre-metabolic activation, improved membrane stability, antioxidant defense, osmolyte accumulation, and ROS metabolism. Additionally, it triggers molecular mechanisms such as the activation of stress-responsive transcription factors, up-regulation of protective proteins, and antioxidant defense genes. These processes contribute to transgenerational effect and epigenetic modifications at the chromatin level, leading to enhanced plant adaptation to environmental stresses caused by climate change. This stress memory is achieved through transcriptional, translational, and epigenetic modifications, leading to sustained stress tolerance. Furthermore, seed priming positively impacts soil microbiomes by improving root structure, nutrient uptake, and microbial diversity, ultimately promoting sustainable agricultural practices. Created in BioRender. Gohari, G. (2025) https://BioRender.com/i99t835.

Importantly, priming induces stress memory via seed or plant exposure to a mild stress level, which enables them to respond more rapidly and effectively to subsequent stress conditions (Fig. 2), keeping the plant in ‘alert mode’ (Johnson and Puthur, 2021). This stress memory is achieved through transcriptional, translational, and epigenetic modifications, leading to sustained stress tolerance (Liu *et al*., 2022). Epigenetic changes include histone modifications (e.g. *H3K27me3* island fractionation), DNA methylation, and chromatin remodeling (Savvides *et al*., 2016; Gohari *et al*., 2024a, b). These modifications enhance stress-responsive gene expression, and stress tolerance can be inherited (Johnson and Puthur, 2021). Importantly, priming enables plants to retain stress memory throughout their life cycle, and transmit it to subsequent generations, promoting transgenerational plasticity (Biswas *et al*., 2023; Jatana *et al*., 2024). DNA methylation and chromatin modifications reinforce stress tolerance and improve plant resilience under environmental challenges (Marthandan *et al*., 2020; Thakur *et al*., 2022; Biswas *et al*., 2023). These inherited epigenetic modifications highlight the role of priming in long-term stress adaptation (Johnson and Puthur, 2021; Gohari *et al*., 2024a, b).

Molecular changes after priming contribute to preservation of ion homoeostasis, defective DNA repair, early DNA replication, RNA stabilization, higher *de novo* protein synthesis, electrolytic leakage inhibition and up-regulation of gene expression, all important for essential plant processes (e.g. seed germination) (Ibrahim, 2016; Biswas *et al.*, 2023; Marthandan *et al*., 2020; Jatana *et al*., 2024). Priming preserves the damaged chromatin structure through histone post-translational modifications, DNA methylation, and protein glycation by adding reducing sugars to the amino groups in proteins, thereby recovering genome integrity (Thakur *et al*., 2022; Gohari *et al*., 2024a, b). In fact, priming helps DNA repair and replication by sustaining microtubule components and the cytoskeleton. Furthermore, priming exploits molecular chaperones improving plant tolerance to stressors (Thakur *et al*., 2022), and causes epigenetic changes, besides increasing the level of transcription factors and inactive forms of signaling proteins, regulated before imposing stress and then causing a programmed and effective defense mechanism (Ibrahim, 2016; Jatana *et al*., 2024; Gohari *et al*., 2024a, b).

## Conclusions, challenges, and future perspectives

Seed priming leads to significant physio-biochemical and molecular changes in plants, resulting in higher seed germination, seedling emergence, and plant growth and development, particularly under stressful conditions. This technique represents an eco-friendly, inexpensive and sustainable approach to mitigate stress effects and increase yield and nutritional quality, leading to long-term food security. Furthermore, priming safeguards environmental sustainability through a lower need for herbicides and pesticides, while such approaches can fit into the concept of a circular economy, such as through the ‘green’ synthesis of nanoparticles (e.g. ZnO NPs, Fe_3_O_4_ NP) from food waste material (Nile *et al.,* 2022; Wang *et al.,* 2025). Although there has been increasing evidence regarding the beneficial effect of seed priming, further work should be done on commercial agricultural settings in order to account for the effect of multiple and simultaneous stresses representative of ‘real-life’ conditions. Moreover, the effect of environmental conditions on seed storage longevity and priming agents and *vice versa,* particularly in nanodelivery systems, should be assayed to better regulate the method, as sub-optimal application parameters could lead to reduced seed storage capacity and/or shorter seed lifespan, representing a potential bottleneck. Similarly, various priming agents should be investigated under different (biotic and abiotic) stress conditions induced by climate change and human activities, to confirm the best priming approach as a mitigation strategy. This should be strongly linked with the necessity to determine optimal priming agent concentrations and treatment duration in a crop/stress-dependent manner, as this will improve scalability, and thus facilitate the potential commercialization of such technologies. For biopriming approaches in particular, their adoption faces limitations, such as variable efficacy across crops and environments, short shelf-life of microorganisms, incompatibility with some chemical practices, sensitivity to environmental stress, and risk of disease transmission. Additionally, regulatory restrictions, high production and handling costs, and questions of cost-effectiveness can hinder its commercialization and large-scale use.

Caution should be taken for application of NPs, particularly in smart nano delivery systems. The fate and transport of NPs in the environment are critical due to their entrance to soil, water, and air over several routes that cause their accumulation in food chains to evaluate their long-term impacts in order to assess and address health and safety concerns. The safe use of the system demands appropriate protocols based on comprehensive research in agriculture and other sectors. Equally, their production and application require the establishment of the appropriate legal frameworks. Likewise, their fate and possible toxicity in the environment should be assayed and determined due to the connectivity of agricultural activities with many ecosystems. This emphasizes the importance of producing safer NPs with better physico-chemical properties for seed protection, biofortification, plant resistance and tolerance against pests and abiotic stresses, or even the mix of these effects. Moreover, the need for lower concentrations of priming compounds in a nanodelivery system effectively results in avoiding high release of the materials in the environment (that could lead to contamination of soil and water resources) and, thus, lower residual amounts in plants, thus necessitating the development of sustainable, effective and standardized protocols. Accordingly, since fundamental knowledge is still relatively fragmentary, complete surveys are mandated to understand the commonly controlled networks following NP application, in order to elucidate their mode of action.
